# Essential roles of aspartate aminotransferase 1 and vesicular glutamate transporters in β-cell glutamate signaling for incretin-induced insulin secretion

**DOI:** 10.1371/journal.pone.0187213

**Published:** 2017-11-01

**Authors:** Naoya Murao, Norihide Yokoi, Kohei Honda, Guirong Han, Tomohide Hayami, Ghupurjan Gheni, Harumi Takahashi, Kohtaro Minami, Susumu Seino

**Affiliations:** 1 Division of Molecular and Metabolic Medicine, Department of Physiology and Cell Biology, Kobe University Graduate School of Medicine, Kobe, Japan; 2 Kansai Electric Power Medical Research Institute, Kobe, Japan; 3 Division of Medical Chemistry, Department of Biophysics, Kobe University Graduate School of Health Sciences, Kobe, Japan; 4 Division of Diabetes, Department of Internal Medicine, Aichi Medical University, Nagakute, Japan; Baylor College of Medicine, UNITED STATES

## Abstract

Incretins (GLP-1 and GIP) potentiate insulin secretion through cAMP signaling in pancreatic β-cells in a glucose-dependent manner. We recently proposed a mechanistic model of incretin-induced insulin secretion (IIIS) that requires two critical processes: 1) generation of cytosolic glutamate through the malate-aspartate (MA) shuttle in glucose metabolism and 2) glutamate transport into insulin granules by cAMP signaling to promote insulin granule exocytosis. To directly prove the model, we have established and characterized CRISPR/Cas9-engineered clonal mouse β-cell lines deficient for the genes critical in these two processes: aspartate aminotransferase 1 (AST1, gene symbol *Got1*), a key enzyme in the MA shuttle, which generates cytosolic glutamate, and the vesicular glutamate transporters (VGLUT1, VGLUT2, and VGLUT3, gene symbol *Slc17a7*, *Slc17a6*, and *Slc17a8*, respectively), which participate in glutamate transport into secretory vesicles. *Got1* knockout (KO) β-cell lines were defective in cytosolic glutamate production from glucose and showed impaired IIIS. Unexpectedly, different from the previous finding that global *Slc17a7* KO mice exhibited impaired IIIS from pancreatic islets, β-cell specific *Slc17a7* KO mice showed no significant impairment in IIIS, as assessed by pancreas perfusion experiment. Single *Slc17a7* KO β-cell lines also retained IIIS, probably due to compensatory upregulation of *Slc17a6*. Interestingly, triple KO of *Slc17a7*, *Slc17a6*, and *Slc17a8* diminished IIIS, which was rescued by exogenously introduced wild-type *Slc17a7* or *Slc17a6* genes. The present study provides direct evidence for the essential roles of AST1 and VGLUTs in β-cell glutamate signaling for IIIS and also shows the usefulness of the CRISPR/Cas9 system for studying β-cells by simultaneous disruption of multiple genes.

## Introduction

Insulin, a key hormone regulating glucose metabolism, is secreted from pancreatic β-cells to maintain blood glucose levels within a normal range. Glucose is physiologically the most important substance that regulates insulin secretion. Glucose-induced insulin secretion (GIIS) comprises two pathways: the triggering pathway and the metabolic amplifying pathway [1]. The former involves closure of the ATP-sensitive K^+^ channels by increased ATP concentration due to glucose metabolism, which is followed by Ca^2+^ influx through voltage-dependent Ca^2+^ channels leading to insulin granule exocytosis. The latter involves amplification of the effects of the triggering pathway by metabolic signals other than ATP evoked by glucose metabolism, but the mechanism remains poorly understood. In addition to glucose, neuro-hormonal amplification is critical in normal regulation of insulin secretion, as reviewed in detail by Yokoi et al. [2]. Hormones and neurotransmitters exert their effects on insulin secretion mainly through G-protein coupled receptor-mediated signals such as cAMP, diacylglycerol, and inositol 1,4,5-triphosphate. The incretins glucagon-like peptide 1 (GLP-1) and glucose-dependent insulinotropic polypeptide (GIP), which are secreted from enteroendocrine cells in response to meal ingestion, amplify insulin secretion through cAMP signaling in pancreatic β-cells [3,4]. Although the effects of incretins in insulin secretion are glucose concentration-dependent [5–7], the interaction between glucose metabolism and incretin/cAMP signaling was not known. We recently found β-cell glutamate acts as a critical signal in incretin-induced insulin secretion (IIIS) and proposed a mechanistic model of β-cell glutamate signaling in IIIS, which requires both production of cytosolic glutamate through the malate-aspartate (MA) shuttle linked to glycolysis and transport of cytosolic glutamate into insulin granules via cAMP signaling [2,8]. However, this model was based primarily on experiments using gene knockdown and/or pharmacological inhibition of aspartate aminotransferase 1 (AST1), an enzyme in the MA shuttle that generates cytosolic glutamate by transferring amino residue from aspartate to α-ketoglutarate, and vesicular glutamate transporter VGLUT1, which mediates glutamate transport into insulin granules [9–12].

The recently developed RNA-guided CRISPR (clustered regularly interspaced short palindromic repeat)/Cas9 genomic editing system has made it possible to selectively disrupt any gene of interest [13]. This technology is now widely used for simple and rapid establishment of gene deficient animals and cell lines. However, there are few reports on its application to pancreatic β-cell lines [14]. In the present study, we applied CRISPR/Cas9 technology to directly prove our model of IIIS by establishing and characterizing CRISPR/Cas9-engineered clonal mouse β-cell lines deficient for the genes involved in glutamate signaling: AST1 (*Got1*), VGLUT1 (*Slc17a7*), VGLUT2 (*Slc17a6*), and VGLUT3 (*Slc17a8*).

## Materials and methods

### Cell lines

MIN6-K8, a mouse pancreatic β-cell line that secretes insulin in response to both glucose and incretins, was established from the IT6 mice as described previously [15].

### Cell culture

MIN6-K8 and gene deficient β-cell lines were cultured in Dulbecco’s modified Eagle’s medium (DMEM) containing 10% heat-inactivated fetal bovine serum and maintained in a humidified incubator with 95% air and 5% CO_2_ at 37°C.

### CRISPR/Cas9 transfection and clonal isolation of cells

The Cas9 D10A nickase expression vector and sgRNA vectors containing hygromycin resistant markers were purchased from GeneCopoeia (Rockville, MD, USA). MIN6-K8 cells were reverse-transfected with a combination of the Cas9 nickase expression vector and a pair of L/R-sgRNA vectors (0.133 μg/mL for each) using Lipofectamine 2000 transfection reagent (Life Technologies, Carlsbad, CA, USA) according to the manufacturer’s instruction. After 48 hours of incubation, 400 μg/mL hygromycin B was added for selection. After 4 days of incubation, cells were re-plated to recover for 11 days. For clonal isolation, cells were serially diluted in 10 cm-dishes to form single colonies, which were then manually picked under microscopy and isolated in a 48 well-plate.

### Sequencing

Genomic DNA was extracted from each colony using SimplePrep reagent for DNA (Takara Bio, Otsu, Japan) according to the manufacturer’s instruction. Cas9 target regions were amplified by PCR and the PCR products were subjected to agarose gel electrophoresis. The PCR products showing different length from the wildtype allele were cloned into pMD20-T blunt vector (Takara Bio, Otsu, Japan) and analyzed by Sanger sequencing using 3100-Avant Genetic Analyzer (Applied Biosystems, Foster City, CA, USA).

### Insulin secretion from cultured β-cell lines

MIN6-K8 and gene knockout β-cell lines were preincubated for 30 min in HEPES-balanced Krebs-Ringer bicarbonate buffer containing 0.1% BSA (H-KRB) with 2.8 mM glucose, then incubated for 30 min in H-KRB with 2.8 mM glucose, 16.7 mM glucose, 16.7 mM glucose plus GLP-1 or GIP, or 16.7 mM glucose plus dimethyl glutamate (dmGlu). Insulin released in the incubation buffer and cellular insulin content were measured by homogeneous time-resolved fluorescence (HTRF) assay using insulin assay kits from CIS Bio international (Gif sur Yvette, France). The amounts of insulin secretion were normalized by the cellular insulin content determined after extraction by 0.1% Triton-X.

### RT-PCR

Total RNA was isolated from cultured β-cell lines or mouse pancreatic islets using QIAshredder and RNeasy mini kit (Qiagen, Hilden, Germany). cDNA was synthesized by reverse transcription of total RNA using a ReverTraAce qPCR RT Kit (Toyobo, Osaka, Japan). PCR of cDNA fragment harboring Cas9 target region was performed by using AmpliTaq Gold 360 Master Mix (Applied Biosystems) and GeneAmp PCR system 9700 thermal cycler (Applied Biosystems). Primer sets: *Slc17a7*, allele 1 (forward: GCAGGAGGAGTTTCGGAAG, reverse: GTGGGTTGTGCTGTTGTTGA), allele 2 (forward: GCAGGAGGAGTTTCGGAAG, reverse: CCATGTATGAGGCCGACAGT), *Slc17a6* (forward: ACTATGCGCAGAATCCGTCT, reverse: CCTGGAATCTGGGTGATGAT), *Slc17a6* V61 allele 1 (forward: CTCCGCTATGCGACTGTGCT, reverse: CCTGGAATCTGGGTGATGAT), *Slc17a6* V61 allele 2 (forward: GTAAGCCCCTGGAGGTCTCA, reverse: CCTGGAATCTGGGTGATGAT), and *Got1* (forward: AGGTCTCGGCACATTCTGTC, reverse: TGAGGCTGTTGTCGTTAGCA). mRNA expression levels were determined by quantitative real time PCR using Taqman Gene Expression Assays, Taqman Gene Expression Master Mix II, with UNG (Applied Biosystems) and a StepOnePlus real-time PCR system (Applied Biosystems). Relative abundance of mRNAs was calculated by ΔΔCT and normalized to endogenous *Gapdh* or *Rn18s* mRNA as invariant controls. Probe sets: Mm00494693_m1 (*Got1*), Mm00499876_m1 (*Slc17a6*), Mm00812886_m1 (*Slc17a7*), Mm00805413_m1 (*Slc17a8*), Mm99999915_g1 (*Gapdh*), and Mm03928990_g1 (*Rn18s*).

### Western blotting

Cells were homogenized with lysis buffer [50 mM Tris-HCl (pH7.5), 150 mM NaCl, 1% NP40, 0.5% sodium deoxycholate, 0.1% SDS, and protease inhibitors (cOmplete Protease Inhibitor Cocktail, Roche, Basel, Switzerland)]. Proteins were separated by SDS-PAGE and electrophoretically transferred onto a PVDF membrane (Immobilon P, Millpore, Billerica, MA, USA). The membranes were blocked in blocking solution (EzBlock Chemi, Atto, Tokyo, Japan), incubated with anti-GOT1 antibody (Sigma, St. Louis, MO, USA) for 1h, washed with tris-buffered saline [20 mM Tris-HCl (pH7.5), 150 mM NaCl] containing 0.1% Tween 20 (TBS-T), incubated with HRP-conjugated secondary antibodies for 1h, and washed with TBS-T. Immunoreactivity was visualized with an enhanced chemiluminescence system, ECL Prime detecting reagents (GE Healthcare, Little Chalfont, UK) and detected by ImageQuant LAS 4000mini (GE Healthcare).

### Rescue experiment by overexpression of wild type (WT) genes

The pcDNA3.1(+) N-DYK empty vector, pcDNA3.1(+) N-DYK vectors containing cDNA of *Got1* or *INS1*, pcDNA3.1(+) vectors containing cDNA of *Slc17a7* or *Slc17a6* were purchased from Genscript (Piscataway, NJ, USA). *Got1* KO β-cell lines were reverse-transfected with a combination of *INS1* (0.4 μg/mL) and either *Got1* (0.46 μg/mL) or empty pcDNA3.1(+) N-DYK construct (control, 0.46 μg/mL). VGLUTs (*Slc17a7*, *Slc17a6*, and *Slc17a8*) triple KO β-cell lines were reverse-transfected with either *INS1* (0.4 μg/mL, control) or a combination of *INS1* (0.4 μg/mL) and *Slc17a7* (0.0025 μg/mL) or *Slc17a6* (0.0025 μg/mL). For transfection, Lipofectamine 2000 transfection reagent (Life Technologies) was used according to the manufacturer’s instruction. After 48 hours of incubation, human C-peptide secretion experiment was performed in the same manner as in the insulin secretion experiment. Human C-peptide released in the incubation buffer and cellular C-peptide content were measured by Ultrasensitive C-peptide ELISA (Mercodia, Uppsala, Sweden).

### Measurement of glutamate content

MIN6-K8 and *Got* KO β-cell lines were preincubated for 60 min in H-KRB with 2.8 mM glucose and then incubated for another 30 min in H-KRB with [U-^13^C]-glucose (2.8 mM or 16.7 mM). Extraction and quantification of cytosolic glutamate contents was performed as previously described [8]. Glutamate contents were normalized by protein contents determined by BCA protein assay kit (Thermo Scientific,Waltham, MA).

### Mice

The *Slc17a7*-floxed mice with a floxed allele, in which exon 2 of *Slc17a7* was sandwiched by loxP, were established. The *Slc17a7*-floxed mice were then crossbred with Ins-Cre mice that express β-cell specific Cre [16] to produce β-cell specific *Slc17a7* knockout (β*Slc17a7*^−/−^) mice. The *Slc17a7*^flox/flox^ mice were used as control. All animals were maintained under specific pathogen free conditions at 23 ± 2°C and 55 ± 10% relative humidity with a 12-h light-dark cycle, and were provided with water and a commercial diet CE-2 (CLEA Japan, Inc., Tokyo, Japan) at the Animal Facility of Kobe Biotechnology Research and Human Resource Development Center of Kobe University. At the end of the experiments, animals were sacrificed by cervical dislocation or overdose of anesthesia with pentobarbital sodium. All animal experiments were approved by the Committee on Animal Experimentation of Kobe University, and carried out in accordance with the Guidelines for Animal Experimentation at Kobe University. The *Slc17a7*-floxed mice are available from the RIKEN BRC (http://mus.brc.riken.jp/, RBRC09669).

### Immunofluorescence staining of mouse pancreas

Mouse pancreata were fixed and pretreated as previously described [17]. Tissues were incubated with guinea pig anti-insulin antibody (ab7842, Abcam, Tokyo, Japan) (1:100) and rabbit anti-VGLUT1 antibody (a gift from Y. Moriyama [18]) (1:100) overnight at 4°C, followed by Alexa Fluor 488-conjugated goat anti-guinea pig IgG antibody (A-11073, Molecular Probes, Eugene, Oregon, USA) (1:500) and Alexa Fluor 546-conjugated goat anti-rabbit IgG antibody (A-11035, Molecular Probes, Eugene, Oregon, USA) (1:500) in a dark chamber for 1.5 hours at room temperature, respectively. Nuclei were visualized by 4',6-diamidino-2-phenylindole (DAPI, Dojindo, Kumamoto, Japan) (1:2000). The immunostained cells were observed with a BZ9000 microscope (Keyence, Osaka, Japan).

### Immunofluorescence staining of cultured β-cell lines

MIN6-K8, VGLUT1 (*Slc17a7*) single KO, and VGLUTs (*Slc17a7*, *Slc17a6*, and *Slc17a8*) triple KO cell lines were fixed with 3.7% formaldehyde in 0.1 M phosphate buffered saline (PBS) adjusted to pH 7.4 for 10 min at room temperature and thoroughly rinsed with 0.1 M PBS. After pretreatment with 10% normal goat serum, cells were incubated with rabbit anti-insulin (H-86) antibody (sc-9168, Santa Cruz Biotechnology, Dallas, Texas, USA) and guinea-pig anti-VGLUT1 antibody (AB5905, Millipore, Darmstadt, Germany) (1:5000) or guinea-pig anti-VGLUT2 antibody (AB2251-I, Millipore) (1:500) overnight at 4°C, followed by Alexa Fluor 488-conjugated goat anti rabbit IgG antibody (A-11008, Molecular Probes) (1:500) and Alexa Fluor 594-conjugated goat anti-guinea pig IgG antibody (A-11076, Molecular Probes) (1:500) in a dark chamber for 1.5 hours at room temperature, respectively. Cells were treated with DAPI and observed under microscopy as described above.

### Pancreas perfusion experiment

Pancreas perfusion experiments were performed as previously described [19]. Overnight (16 h) fasted male mice at 17–18 weeks of age were used. Briefly, the perfusion protocol began with a 20-min equilibration period with the same buffer used in the initial step shown in the figures. The flow rate of the perfusate was 1 mL/min. The insulin levels in the perfusate were measured by using the HTRF assay as described above.

### Statistical analysis

The results are presented as mean ± SEM. Differences among the groups were analyzed with Dunnett’s method or Welch’s t-test as indicated in the legends. p < 0.05 was regarded as statistically significant.

## Results and discussion

### Establishment and characterization of AST1 (*Got1*) knockout β-cell lines

We first focused on AST1 (gene symbol *Got1*), the enzyme in the MA shuttle that produces cytosolic glutamate in β-cells. We applied the CRISPR/Cas9 nickase system [20], a modification of the CRISPR/Cas9 system with improved specificity, for disrupting *Got1* in MIN6-K8. In the CRISPR/Cas9 nickase system, a combination of Cas9 nickase and a pair of sgRNAs induces nicks on both DNA chains to mediate double-stranded breaks (DSB), which is repaired by nonhomologous end joining (NHEJ) with resultant indels [20]. Targeting the genomic locus by two sgRNAs designed for each DNA chain can minimize off-target cleavage [21]. To avoid functional truncated products, the target sequence was set within the first exon. The MIN6-K8 β-cell line was transfected with three plasmids expressing Cas9 nickase and a pair of sgRNAs. Cells were selected with hygromycin and cultured in a 10 cm plate in a very low concentration to form single colonies, the each of which was then isolated and cultured separately to yield different colonies.

**Fig 1:**
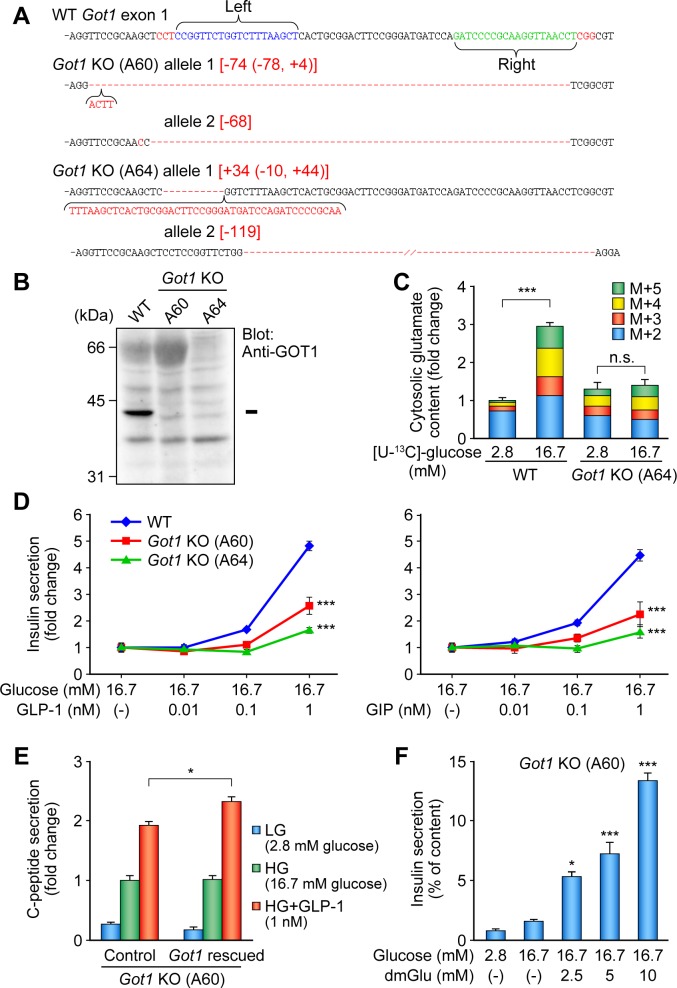
Establishment and characterization of *Got1* KO β-cell lines. (A) Mutations in *Got1* exon 1 in two *Got1* KO cell lines induced by the CRISPR/Cas9 nickase system. WT sequence is shown with target sites of sgRNAs. Protospacer adjacent motif (PAM) and mutations are shown in red. (B) Absence of AST1 protein in *Got1* KO cell lines revealed by western blotting. (C) Cytosolic glutamate content in *Got1* KO cell line. WT MIN6-K8 or *Got1* KO (A64) cell lines were stimulated with [U-^13^C]-glucose and ^13^C-enriched glutamate isotopomers M+2 to M+5 (two to five substitutions of ^12^C by ^13^C) were quantified by mass spectrometry (n = 3). (D) Insulin secretory response in *Got1* KO cell lines. The cell lines were stimulated with glucose and incretin (GLP-1 or GIP) (n = 4). Insulin secretion was normalized by cellular insulin content and presented as fold-change relative to the amount of insulin secretion at 16.7 mM glucose. (E) Rescue of the AST1 activity by introducing WT *Got1* into *Got1* KO cell line. The *Got1* KO (A60) cell line was transfected with *INS1* along with *Got1* or empty construct and stimulated with glucose and GLP-1 (n = 4). C-peptide secretion was normalized by cellular C-peptide content and the data are presented as fold-change relative to the amount of C-peptide secretion at 16.7 mM glucose. (F) The effect of dimethyl glutamate (dmGlu) on insulin secretion. The *Got1* KO (A60) cell line was stimulated with glucose and dmGlu (n = 4). Insulin secretion was normalized by cellular insulin content. The data are expressed as means ± SEM. Representative results are shown (C, D, E, and F). Similar results were found in 3 independent experiments. Welch’s t-test was used for evaluation of statistical significance vs. 2.8 mM glucose in (C) and vs. control in (E). Dunnett's method was used for evaluation of statistical significance vs. WT in (D) and vs. 16.7 mM glucose in (F). *p < 0.05; ***p < 0.001; n.s., not significant.

We next confirmed mutations in the target region of isolated colonies. First, we amplified the Cas9 target region of each colony by PCR and examined the length of the PCR product for differences from wild-type (WT) allele. To verify the mutation on each allele separately, the PCR products were cloned into pMD20-T vector, and multiple transformants for each colony were sequenced. As a result, two different *Got1* knockout (KO) β-cell lines, A60 and A64, were established, both of which were compound heterozygous mutants having frameshifts on both alleles (Fig 1A). Protein expression of AST1 was completely abolished in both KO cell lines (Fig 1B). To rule out contamination by WT cell line, the absence of WT mRNA was confirmed by PCR of cDNA fragments harboring the Cas9 target region (S1 Fig). qRT-PCR analysis using a Taqman probe targeting 3’ region of the gene showed that the expression level of *Got1* was significantly down-regulated in these KO cell lines (S2 Fig).

To learn if cytosolic glutamate production is impaired by *Got1* deficiency, we measured cytosolic glutamate contents by metabolic flux analysis using stable isotope-labelled [U-^13^C]-glucose. M and M+1 glutamate isotopomers are both naturally existing glutamate in cells, while M+2, M+3, M+4, and M+5 glutamate isotopomers are synthesized *de novo* from [U-^13^C]-glucose. We found that M+2 to M+5 glutamate isotopomers were significantly increased by glucose stimulation in WT cell line, whereas they were not increased in *Got1* KO cell line (Fig 1C), indicating that AST1 is essential for cytosolic glutamate production from glucose.

We then examined insulin secretory response in *Got1* KO cell lines. It is known that insulin secretory ability varies among different β-cell lines. Since we found that the amount of released insulin varied between *Got1* KO (A60 and A64) cell lines established (S3 Fig), incretin responsiveness was evaluated by expressing the insulin secretion data as fold increase relative to the amount of insulin secretion at 16.7 mM glucose. Both *Got1* KO cell lines exhibited a significant impairment in insulin secretion in response to both GLP-1 and GIP, as compared to WT MIN6-K8 cell line (Fig 1D and S3 Fig). GIIS was only mildly impaired in *Got1* KO cell lines (S3 Fig)

To verify that AST1 is required for IIIS, we next tried to rescue the diminished AST1 activity by introducing WT *Got1* into *Got1* KO cell line. Due to low transfection efficiency in MIN6-K8 β-cell line, we performed co-transfection assay with WT *Got1* and human preproinsulin cDNA [22]. Proinsulin is converted into insulin and C-peptide during the secretory process. Since antibody against human C-peptide does not cross-react with endogenous mouse C-peptide, the released human C-peptide can be monitored as an indicator of insulin secretion from the transfected cells. Transfection of *Got1* KO (A60) cell line with WT *Got1* significantly restored incretin-induced C-peptide secretion (Fig 1E). We also examined the effect of dimethyl glutamate (dmGlu), a membrane permeable glutamate precursor [23] that is converted to glutamate in insulin granules as well as in cytosol [8]. In *Got1* KO (A60) cell line, dmGlu amplified insulin secretion in a dose-dependent manner (Fig 1F), indicating that dmGlu complements glutamate deficiency in the *Got1* KO cell line and that cellular glutamate acts as an amplifying signal in insulin secretion.

### Establishment and characterization of β-cell specific VGLUT1 (*Slc17a7*) knockout mice and *Slc17a7* single knockout β-cell lines

Our previous study showed that knockdown of VGLUT1 (*Slc17a7*) or pharmacological inhibition of glutamate transport reduced insulin secretion in response to incretins while knockdown of VGLUT2 (*Slc17a6*) did not [8]. In addition, there was a significant impairment in IIIS from pancreatic islets of global *Slc17a7* KO mice at pre-weaning two weeks of age [8]. Based on these findings, we initially anticipated that single KO of *Slc17a7* would be enough to cause a severe defect in IIIS.

**Fig 2:**
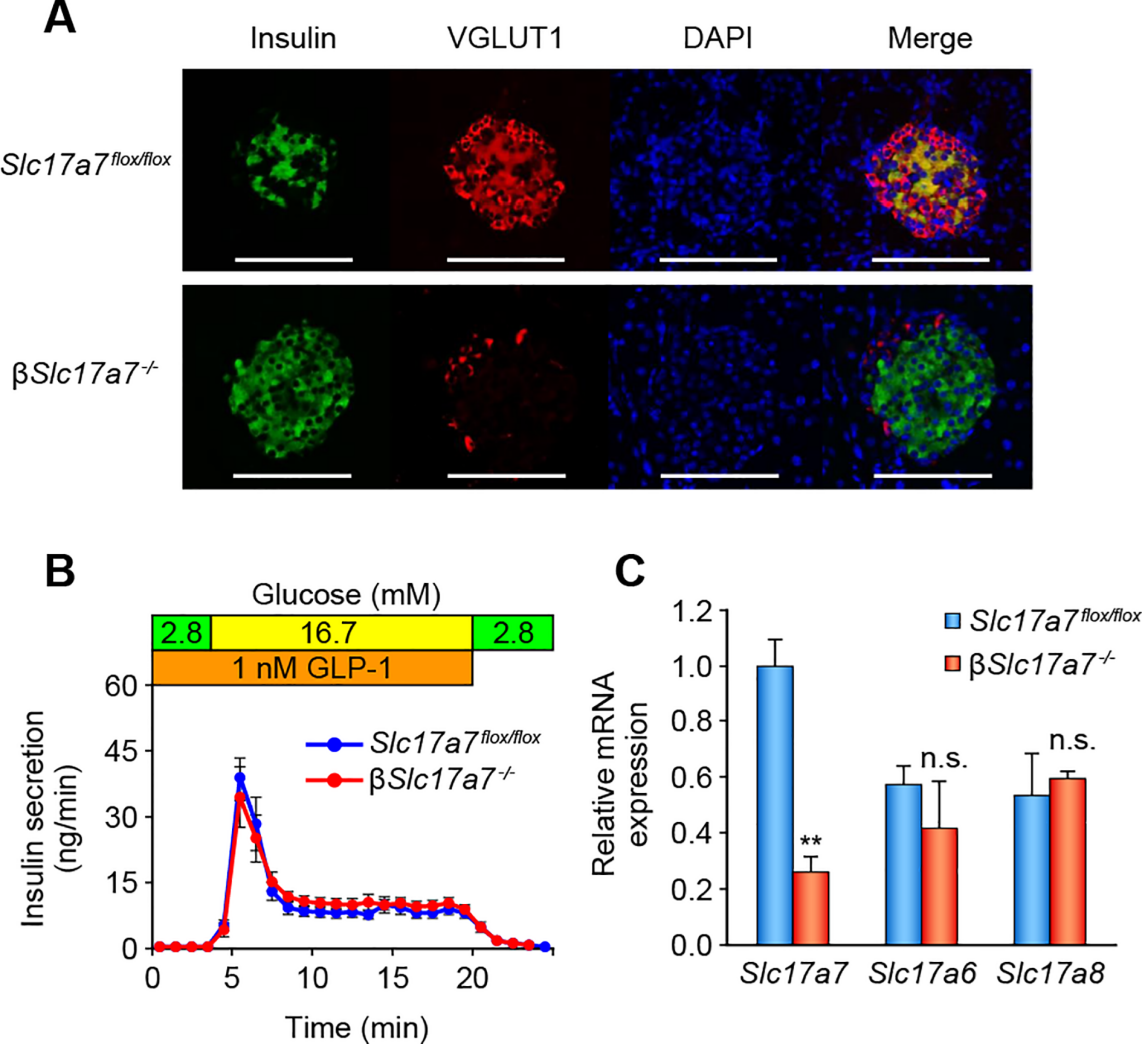
Characterization of β-cell specific *Slc17a7* KO mice. (A) Immunohistochemical analysis of the pancreas of β*Slc17a7*^−/−^ mice. Green, Insulin; red, VGLUT1; blue, 4',6-diamidino-2-phenylindole (DAPI). Scale bars, 100 μm. (B) Pancreas perfusion experiment of *Slc17a7*^*flox*/*flox*^ and β*Slc17a7*^−/−^ mice at 17–18 weeks of age (n = 4). (C) mRNA expression levels of *Slc17a7*, *Slc17a6*, and *Slc17a8* in β*Slc17a7*^−/−^ mice and *Slc17a7*^*flox*/*flox*^ mice (control) at 33 weeks of age (n = 3–4 for each). Welch’s t-test was used for comparisons between the two groups. **p < 0.01; n.s., not significant.

To further ascertain whether VGLUT1 is required for IIIS, we generated β-cell specific *Slc17a7* knockout (β*Slc17a7*^−/−^) mice by using the Cre-loxP system (S4 Fig). The mice showed normal development and there was no gross abnormality. Immunohistochemical staining confirmed that expression of VGLUT1 protein was successfully ablated in pancreatic β-cells of β*Slc17a7*^−/−^ mice (Fig 2A). There were no significant differences in glucose tolerance, meal tolerance, and insulin sensitivity between β*Slc17a7*^−/−^ mice and *Slc17a7*^*flox*/*flox*^ mice (control) (S1 Method and S5 Fig). Unexpectedly, different from our previous finding [8], we found no significant impairment of insulin secretion in response to GLP-1 in β*Slc17a7*^−/−^ mice, as assessed by pancreas perfusion experiment (Fig 2B). RT-PCR analyses of pancreatic islets revealed that although expression level of *Slc17a7* in β*Slc17a7*^−/−^ mice was significantly decreased, there were no significant differences in expression of both *Slc17a6* and VGLUT3 (*Slc17a8*) between β*Slc17a7*^−/−^ mice and *Slc17a7*^*flox*/*flox*^ mice (Fig 2C). VGLUT3 was reported to be present in membranes of insulin-containing secretory granules [24]. It is possible, therefore, that the expression of *Slc17a6* and/or *Slc17a8* compensates for function of glutamate transport into granules in β*Slc17a7*^−/−^ mice. The discrepancy in the phenotype between global *Slc17a7* KO mice and β-cell specific *Slc17a7* KO mice might be due to the difference in the age of mice: the former were examined at pre-weaning state since the mice could not survive thereafter [25], while the latter were examined at adult state when *Slc17a7* deficiency could be compensated by *Slc17a6* and/or *Slc17a8*.

**Fig 3:**
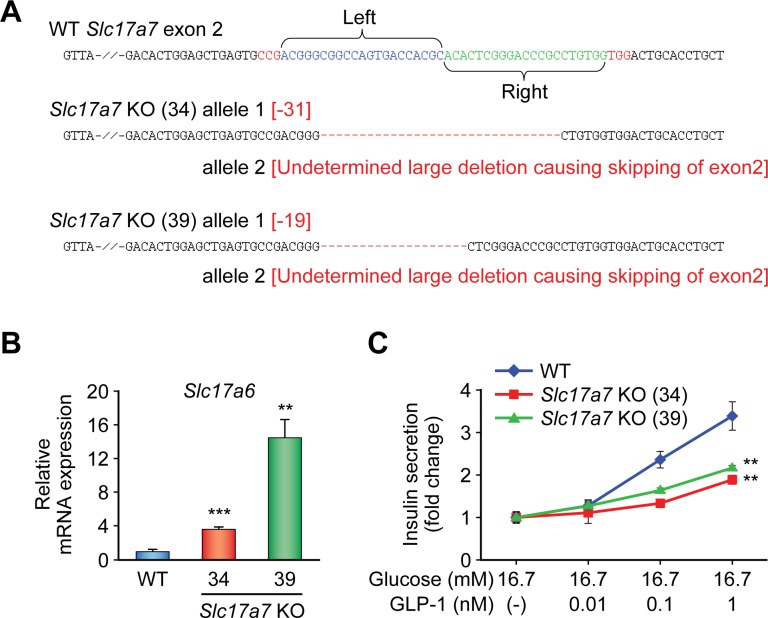
Establishment and characterization of *Slc17a7* single KO β-cell lines. (A) Mutations in *Slc17a7* exon 2 in *Slc17a7* single KO cell lines induced by the CRISPR/Cas9 nickase system. WT sequence is shown with target sites of sgRNAs. Protospacer adjacent motif (PAM) and mutations are shown in red. Allele 2 of both KO cell lines were not detected by PCR probably due to large deletions. (B) mRNA expression levels of *Slc17a6* in *Slc17a7* KO cell lines. The mRNA expression levels of KO cell lines are presented as fold increase relative to those of WT (n = 4). (C) Insulin secretory response in *Slc17a7* single KO cell lines. Cells were stimulated with glucose and GLP-1 (n = 4). Insulin secretion was normalized by cellular insulin content and the data are presented as fold-change relative to the amount of insulin secretion at 16.7 mM glucose. The data are expressed as means ± SEM. Representative results are shown (B and C). Similar results were found in 3 independent experiments. Welch’s t-test with Bonferroni correction was used for evaluation of statistical significance vs. WT in (B). Dunnett's method was used for evaluation of statistical significance vs. WT in (C). **p < 0.01; ***p < 0.001.

**Fig 4:**
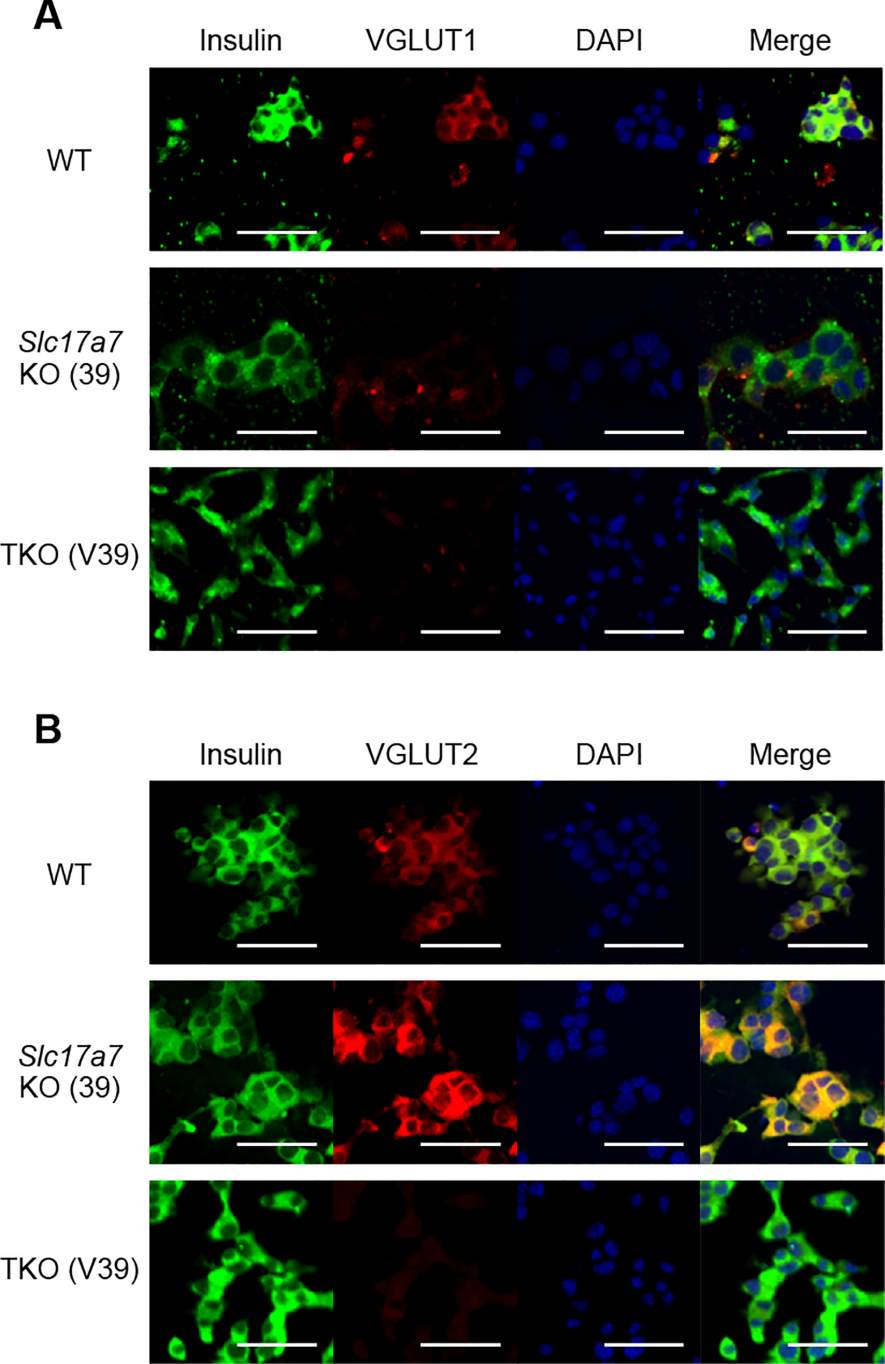
Immunofluorescence staining of *Slc17a7* single KO and *Slc17a7*, *Slc17a6*, and *Slc17a8* triple KO β-cell lines. Green, Insulin; red, VGLUT1 (A) or VGLUT2 (B); blue, 4',6-diamidino-2-phenylindole (DAPI). Scale bars, 50 μm.

In parallel with generation of β*Slc17a7*^−/−^ mice, we also established *Slc17a7* single KO (SKO) β-cell lines. In WT MIN6-K8 cell line, *Slc17a7* and *Slc17a6* are expressed at similar levels and *Slc17a8* is not expressed (S6 Fig). To avoid functional truncated products, exon 2, which encodes the first transmembrane domain of VGLUT1, was Cas9-targeted. Two different SKO cell lines, 34 and 39, were established (Fig 3A). Allele 2 of both clones was not detected by initial PCR-sequencing of genomic DNA, probably due to the large deletions involving the primer sequence. We therefore analyzed the cDNA synthesized from the SKO cell lines 34 and 39. Two different products were amplified by PCR using the cDNA obtained from the cell lines as templates: one corresponding to allele 1 and the other corresponding to allele 2, in which exon 2 is skipped by abnormal splicing due to the large deletions spanning intron 1. The absence of WT *Slc17a7* mRNA was confirmed in both cell lines (S7 Fig). These analyses showed that the SKO cell lines 34 and 39 both are compound heterozygous mutants having frameshifts on both alleles of *Slc17a7*. mRNA expression of *Slc17a7* measured by qPCR was significantly decreased in both SKO cell lines (S8 Fig) and expression of VGLUT1 protein was successfully ablated in the SKO cell line 39, as assessed by immunofluorescence staining (Fig 4A). In contrast, we found that *Slc17a6* was upregulated in SKO cell lines at mRNA and protein levels (Figs 3B and 4B). Expression of *Slc17a8* was not detected. We then examined IIIS in SKO cell lines. Both SKO cell lines retained insulin secretory response to GLP-1 to some extent (Fig 3C and S9 Fig), suggesting that upregulation of VGLUT2 partly compensates for the loss of function of VGLUT1, as was found in β*Slc17a7*^−/−^ mice. Taken together, these findings indicate that VGLUT2 and/or VGLUT3 can compensate for the loss of function of VGLUT1 in IIIS from β-cells. Switching of one VGLUT isoform to another by transcriptional and/or translational regulation was reported in developing mouse cerebellum [26]. Such regulation might also exist in β-cells.

### Establishment and characterization of *Slc17a7*, *Slc17a6*, and *Slc17a8* triple knockout β-cell lines

**Fig 5:**
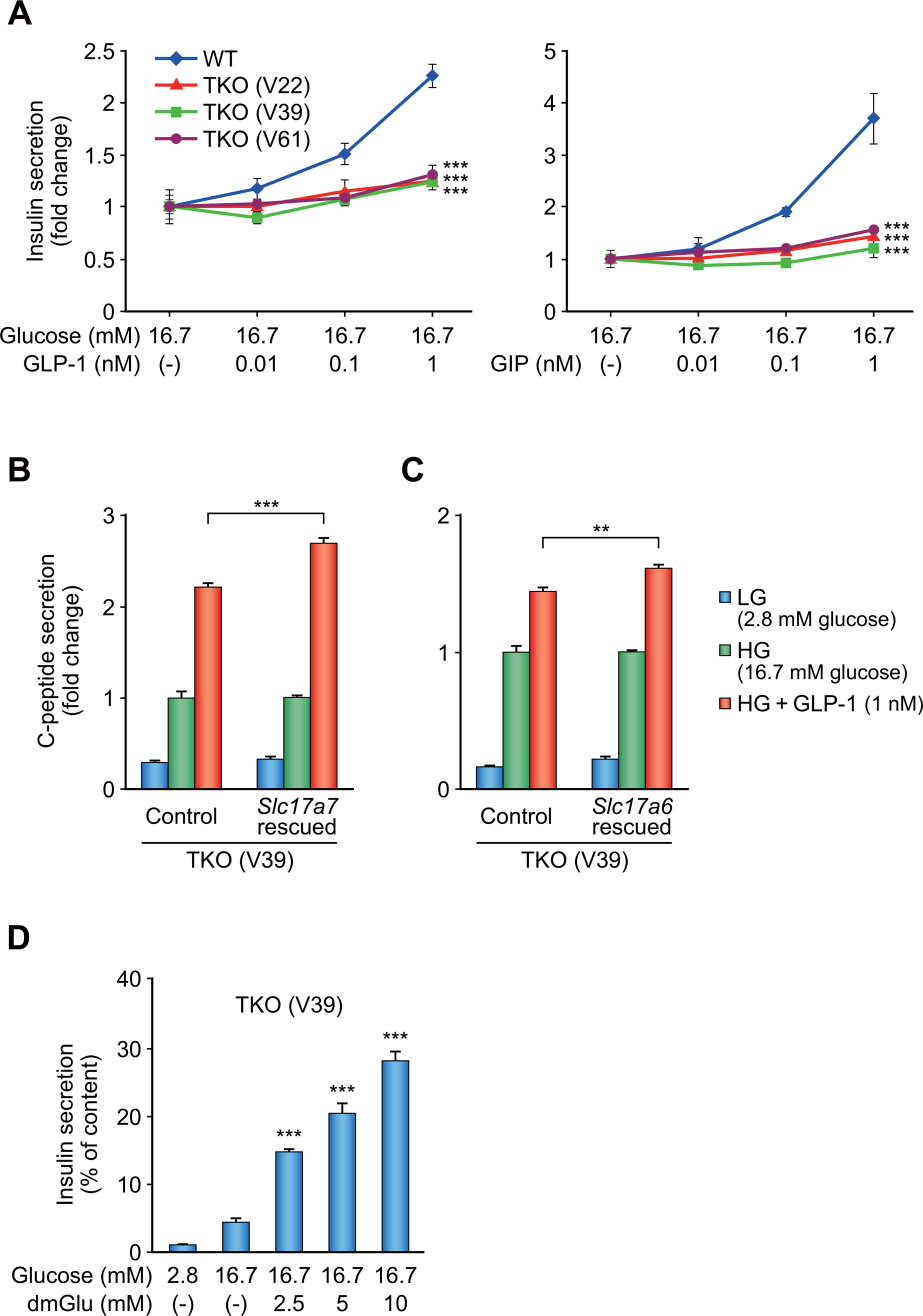
Characterization of *Slc17a7*, *Slc17a6*, and *Slc17a8* triple KO β-cell lines. (A) Insulin secretory response in *Slc17a7*, *Slc17a6*, and *Slc17a8* triple KO (TKO) cell lines. WT and TKO cell lines were stimulated with glucose and incretin (GLP-1 or GIP) (n = 4). Insulin secretion was normalized by cellular insulin content and the data are presented as fold-change relative to the amount of insulin secretion at 16.7 mM glucose. (B, C) Rescue of the VGLUT1 (B) or VGLUT2 (C) activity by introducing WT *Slc17a7* or *Slc17a6* into triple KO cell line, respectively. The cell line V39 was transfected with *INS1* (control) or *INS1* and rescue construct and stimulated with glucose and GLP-1 (n = 4). C-peptide secretion was normalized by cellular C-peptide content and the data are presented as fold-change relative to the amount of C-peptide secretion at 16.7 mM glucose. (D) The effect of dimethyl glutamate (dmGlu) on insulin secretion. The cell line V39 was stimulated with glucose and dmGlu (n = 4). Insulin secretion was normalized by cellular insulin content. The data are expressed as means ± SEM. Representative results are shown. Similar results were found in 3 independent experiments. Dunnett's method was used for evaluation of statistical significance vs. WT in (A) and vs. 16.7 mM glucose in (D). Welch’s t-test was used for evaluation of statistical significance vs. control in (B) and (C). **p < 0.01; ***p < 0.001.

Based on the results above, we generated VGLUT1, VGLUT2, and VGLUT3 (*Slc17a7*, *Slc17a6*, and *Slc17a8*) triple knockout (TKO) β-cell lines. The above-mentioned *Slc17a7* single KO cell line was further transfected with plasmids expressing Cas9 nickase and two pairs of sgRNA each targeting *Slc17a6* and *Slc17a8*, followed by clonal isolation. After confirmation of deficiency of *Slc17a6* in these colonies by sequencing, the Cas9 target region of *Slc17a8* of these colonies were examined. Finally, three TKO cell lines, V22, V39, and V61, were established. As for *Slc17a6*, all of these cell lines were compound heterozygous mutants harboring frameshifts on both alleles (S10 Fig). Absence of WT *Slc17a6* mRNA and VGLUT2 protein was confirmed by PCR of cDNA and immunofluorescence staining, respectively (S11 Fig and Fig 4B). As for *Slc17a8*, both alleles of the TKO cell line V39 and a single allele of the TKO cell line V61 were found to have frameshifts (S10 Fig). Although a single allele of the TKO cell line V22 was found to be in frame, a deletion of 29 amino acids around the first transmembrane domain probably disrupts gene function. Other alleles were not detected by PCR, probably due to the large deletions. We then examined insulin secretory response in these TKO cell lines. GIIS was retained, but IIIS were significantly impaired in all of the three TKO cell lines (Fig 5A and S12 Fig). Exogenous introduction of WT *Slc17a7* or *Slc17a6* into the TKO cell lines significantly rescued incretin-induced C-peptide secretion (Fig 5B and 5C). This finding indicates that VGLUT1 and VGLUT2 functionally compensate each other. We also found that dmGlu markedly amplified insulin secretion in the TKO cell lines (Fig 5D). These findings provide direct evidence that glutamate transport into insulin granules through VGLUTs is essential for IIIS.

## Conclusion

The present study of CRISPR/Cas9-engineered mouse pancreatic β-cell lines provides direct evidence that AST1 and VGLUTs are essential for β-cell glutamate signaling in IIIS. This study is the first to show successful and complete disruption of multiple genes in pancreatic β-cell lines by the CRISPR/Cas9 nickase system. As a family of genes encoding multiple isoforms shares similar functions in general, disruption of one isoform may induce expression of the other to compensate for its function. It is therefore necessary to disrupt multiple target genes simultaneously at cell level to elucidate their functions. The CRISPR/Cas9 system is a useful and straightforward method to disrupt multiple genes simultaneously in pancreatic β-cell lines to elucidate their roles in cellular functions.

## Supporting information

S1 Method Oral glucose tolerance test (OGTT), meal tolerance test (MTT), and intraperitoneal insulin tolerance test (IPITT). For oral glucose tolerance test, glucose (1.5 g/kg) was administered to mice after 6 hours of fasting. For meal tolerance test, 10 μL/g (1.5 g carbohydrate/kg body weight) Twinline (Otsuka Pharmaceuticals, Tokushima, Japan) was administered orally to mice after 6 hours of fasting. For intraperitoneal insulin tolerance test, insulin (0.5 U Humulin R/kg body weight, Eli Lilly Japan K.K., Kobe, Japan) was administered to mice after 6 hours of fasting. Blood glucose levels were measured by a portable glucose meter (ANTSENSE III, HORIBA, Ltd., Kyoto, Japan).
(TIFF)

S1 Fig Absence of WT allele in *Got1* KO cell lines revealed by RT-PCR. Both alleles of the KO cell line A60 and allele 1 of the KO cell line A64 were distinct from the WT allele. Allele 2 of the KO cell line A64 was not detected probably due to low expression.
(TIFF)

S2 Fig Relative mRNA expression levels of *Got1* in *Got1* KO cell lines. mRNA expression levels of KO cell lines are presented as fold-change relative to those of WT (n = 4). The data are expressed as means ± SEM. Representative results are shown. Similar results were found in 3 independent experiments. Dunnett's method was used for statistical comparisons between WT and *Got1* KO cell lines. ***p < 0.001.
(TIFF)

S3 Fig Insulin secretory response in *Got1* KO cell lines. (A, B) Cells were stimulated with glucose and GLP-1 (A) or GIP (B) (n = 4 for each). Insulin secretion was normalized by cellular insulin content. The data are expressed as means ±SEM. Representative results are shown. Similar results are found in 3 independent experiments.
(TIFF)

S4 Fig Targeting strategy for production of the *Slc17a7*-floxed mice. A targeting vector was constructed so that exon 2 coding the first transmembrane domain was flanked by *loxP* sites. The *FRT*-flanked neo gene was excised via Flp-*FRT* recombination. Floxed exon 2 was deleted via Cre-*loxP* recombination.
(TIFF)

S5 Fig Changes in blood glucose levels of β*Slc17a7*^−/−^ mice. Changes in blood glucose levels of control and β*Slc17a7*^−/−^ mice during (A) oral glucose tolerance test, (B) oral meal tolerance test, and (C) intraperitoneal insulin tolerance test. n = 6 each. Differences between the groups were analyzed with the Welch’s t-test. n.s., not significant.
(TIFF)

S6 Fig Relative mRNA expression levels of *Slc17a7*, *Slc17a6*, and *Slc17a8* in WT MIN6-K8 cell lines. mRNA expression levels of *Slc17a6* and *Slc17a8* are presented as fold-change relative to those of *Slc17a7* (n = 3). The data are expressed as means ± SEM. Representative results are shown. Similar results were found in 3 independent experiments. n.d., not detected.
(TIFF)

S7 Fig Absence of WT allele in *Slc17a7* KO cell lines revealed by RT-PCR. Both alleles of *Slc17a7* KO cell lines 34 and 39 were distinct from the WT allele. Detection of allele 1 and 2 required specific primer sets, respectively.
(TIFF)

S8 Fig Relative mRNA expression levels of *Slc17a7* in *Slc17a7* KO cell lines. mRNA expression levels of KO cell lines are presented as fold-change relative to those of WT (n = 4). The data are expressed as means ± SEM. Representative results are shown. Similar results were found in 3 independent experiments. Dunnett's method was used for statistical comparisons between WT and *Slc17a7* KO cell lines. *p < 0.05; ***p < 0.001.
(TIFF)

S9 Fig Insulin secretory response in *Slc17a7* KO cell lines. WT MIN6-K8 and *Slc17a7* single KO (34 and 39) cell lines were stimulated with glucose and GLP-1 (n = 4). Insulin secretion was normalized by cellular insulin content. The data are expressed as means ±SEM. Representative results are shown. Similar results were found in 3 independent experiments.
(TIFF)

S10 Fig Mutations of *Slc17a6* (VGLUT2) and *Slc17a8* (VGLUT3) in VGLUTs triple KO cell lines. (A) Mutations in *Slc17a6* exon 2 in triple KO cell lines induced by the CRISPR/Cas9 nickase system. (B) Mutations in *Slc17a8* exon 2 in triple KO cell lines induced by the CRISPR/Cas9 nickase system. *Slc17a8* allele 2 in cell lines V22 and V61 were not detected by PCR probably due to large deletions. WT sequence is shown with target sites of sgRNAs. PAM and mutations are shown in red.
(TIFF)

S11 Fig The absence of WT *Slc17a6* allele in triple KO cell lines revealed by RT-PCR. (A) Both alleles of TKO cell line V22 and allele 1 of TKO cell line V39 were distinct from the WT allele. Allele 2 of TKO cell line V39 was not detected probably due to low expression. Both alleles of TKO cell line V61 were indistinguishable from the WT allele. (B) Specific primer sets for allele 1 or 2 of TKO cell line V61 proved the mutation.
(TIFF)

S12 Fig Insulin secretory response in triple KO cell lines. (A, B) Cells were stimulated with glucose and GLP-1 (A) or GIP (B) (n = 4 for each). Insulin secretion was normalized by cellular insulin content. The data are expressed as means ±SEM. Representative results are shown. Similar results are found in 3 independent experiments.
(TIFF)
