# Supplementary figures and images for: Essential roles of aspartate aminotransferase 1 and vesicular glutamate transporters in β-cell glutamate signaling for incretin-induced insulin secretion
Source: PLoS One. 2017 Nov 1;12(11):e0187213. doi: 10.1371/journal.pone.0187213 (PMC5665537; doi:10.1371/journal.pone.0187213)

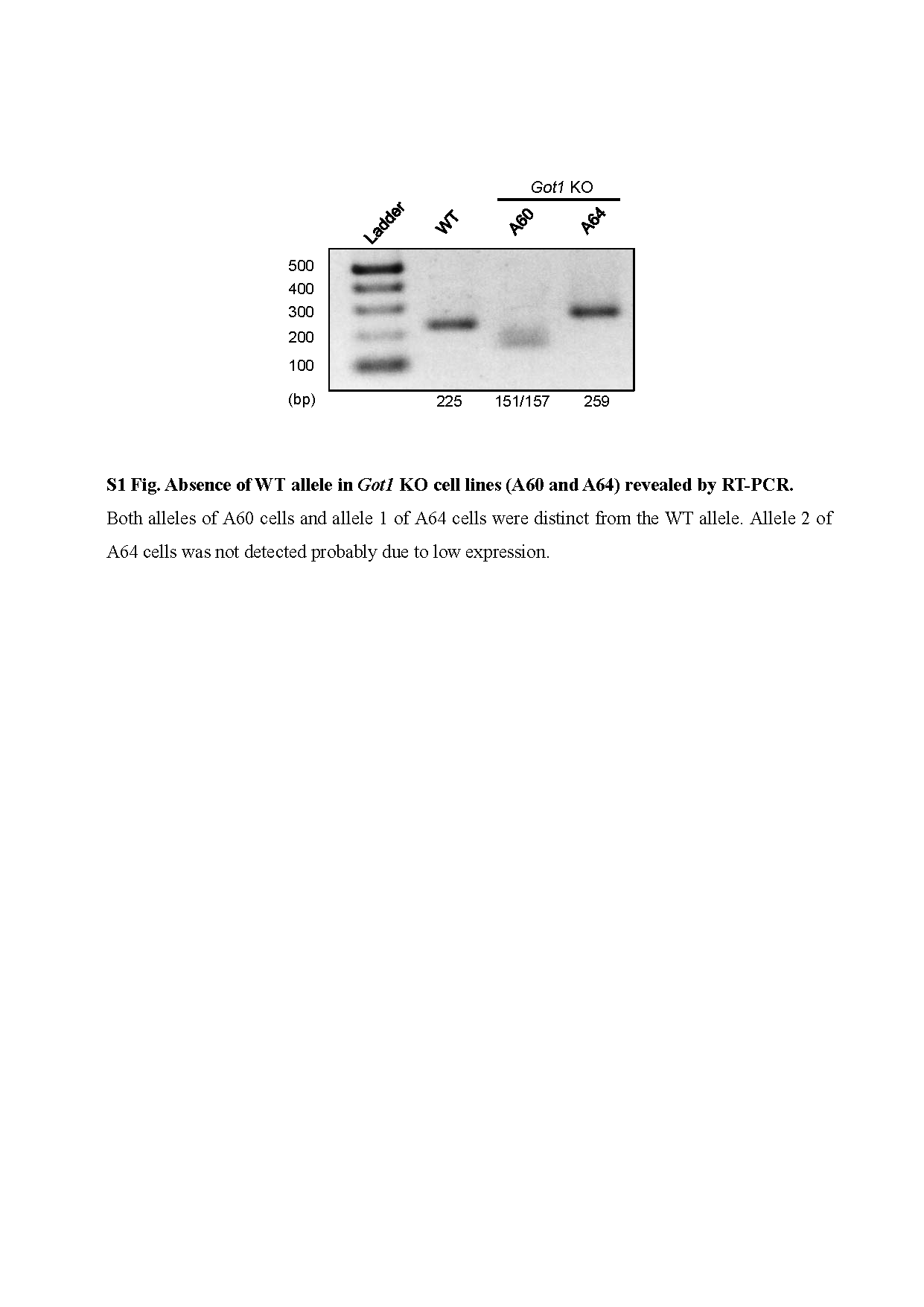

Supplement: S1 Fig — Both alleles of the KO cell line A60 and allele 1 of the KO cell line A64 were distinct from the WT allele. Allele 2 of the KO cell line A64 was not detected probably due to low expression. (TIFF) [file pone.0187213.s002.tiff]

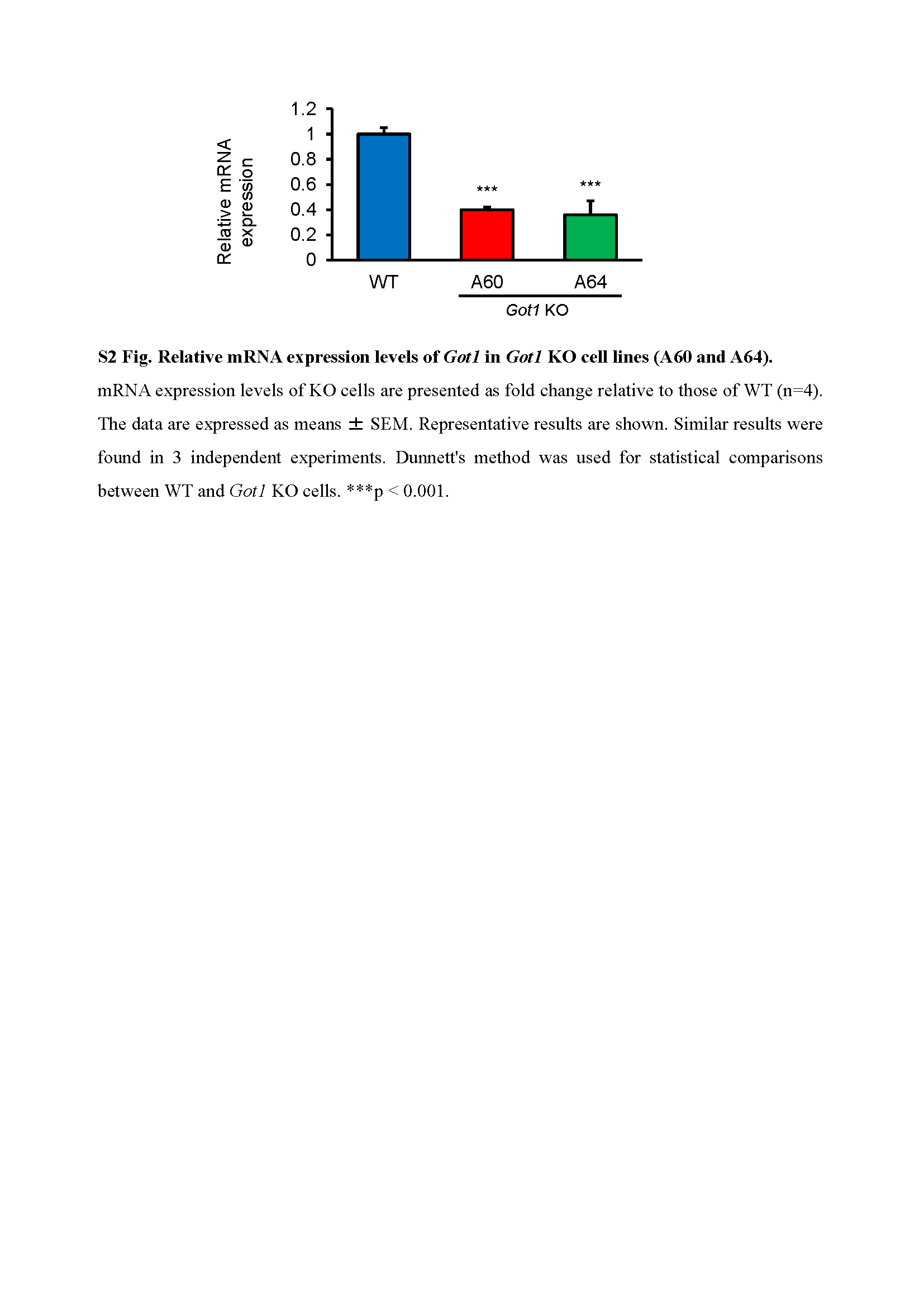

Supplement: S2 Fig — mRNA expression levels of KO cell lines are presented as fold-change relative to those of WT (n = 4). The data are expressed as means ± SEM. Representative results are shown. Similar results were found in 3 independent experiments. Dunnett's method was used for statistical comparisons between WT and Got1 KO cell lines. ***p < 0.001. (TIFF) [file pone.0187213.s003.tiff]

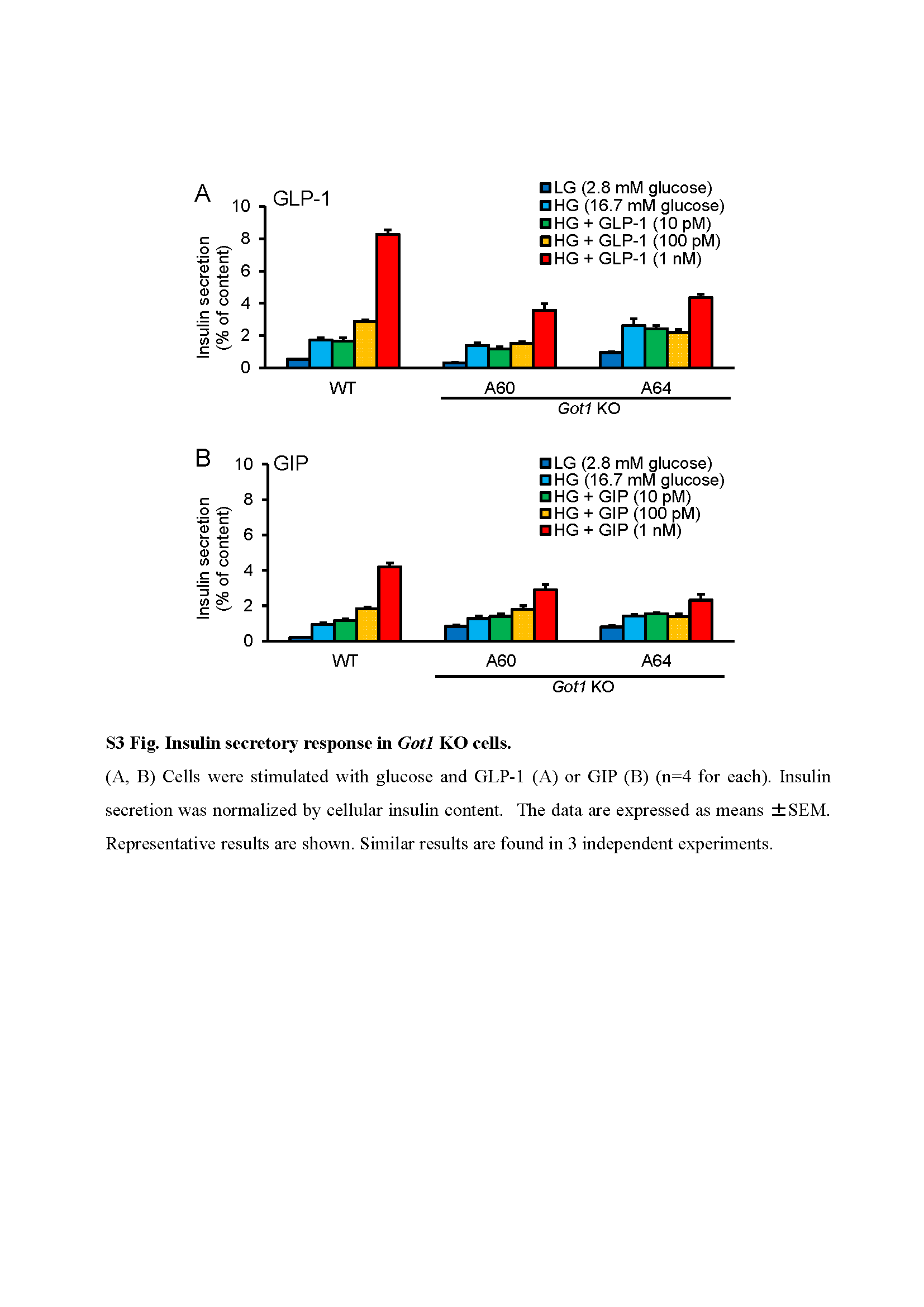

Supplement: S3 Fig — (A, B) Cells were stimulated with glucose and GLP-1 (A) or GIP (B) (n = 4 for each). Insulin secretion was normalized by cellular insulin content. The data are expressed as means ±SEM. Representative results are shown. Similar results are found in 3 independent experiments. (TIFF) [file pone.0187213.s004.tiff]

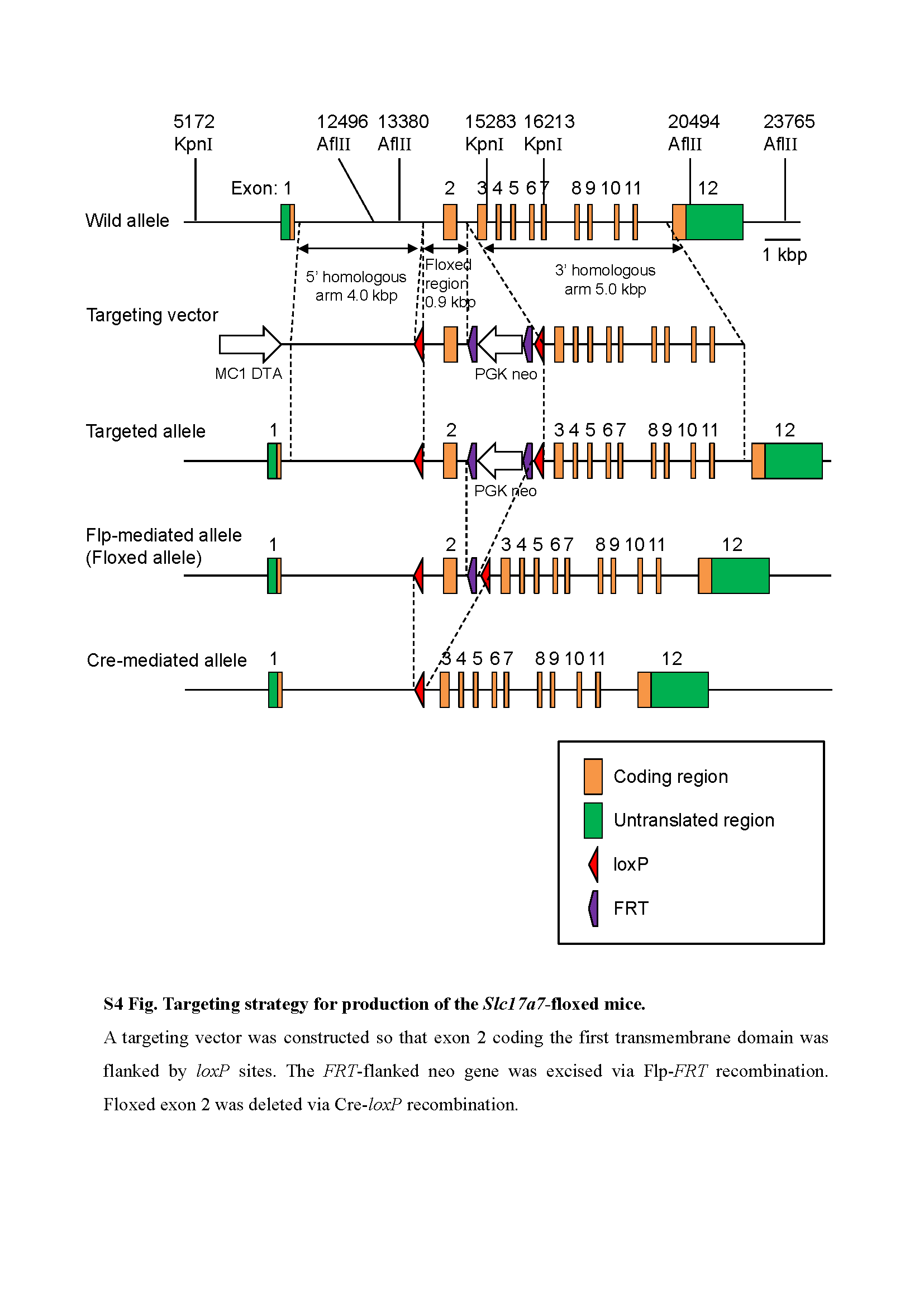

Supplement: S4 Fig — A targeting vector was constructed so that exon 2 coding the first transmembrane domain was flanked by loxP sites. The FRT-flanked neo gene was excised via Flp-FRT recombination. Floxed exon 2 was deleted via Cre-loxP recombination. (TIFF) [file pone.0187213.s005.tiff]

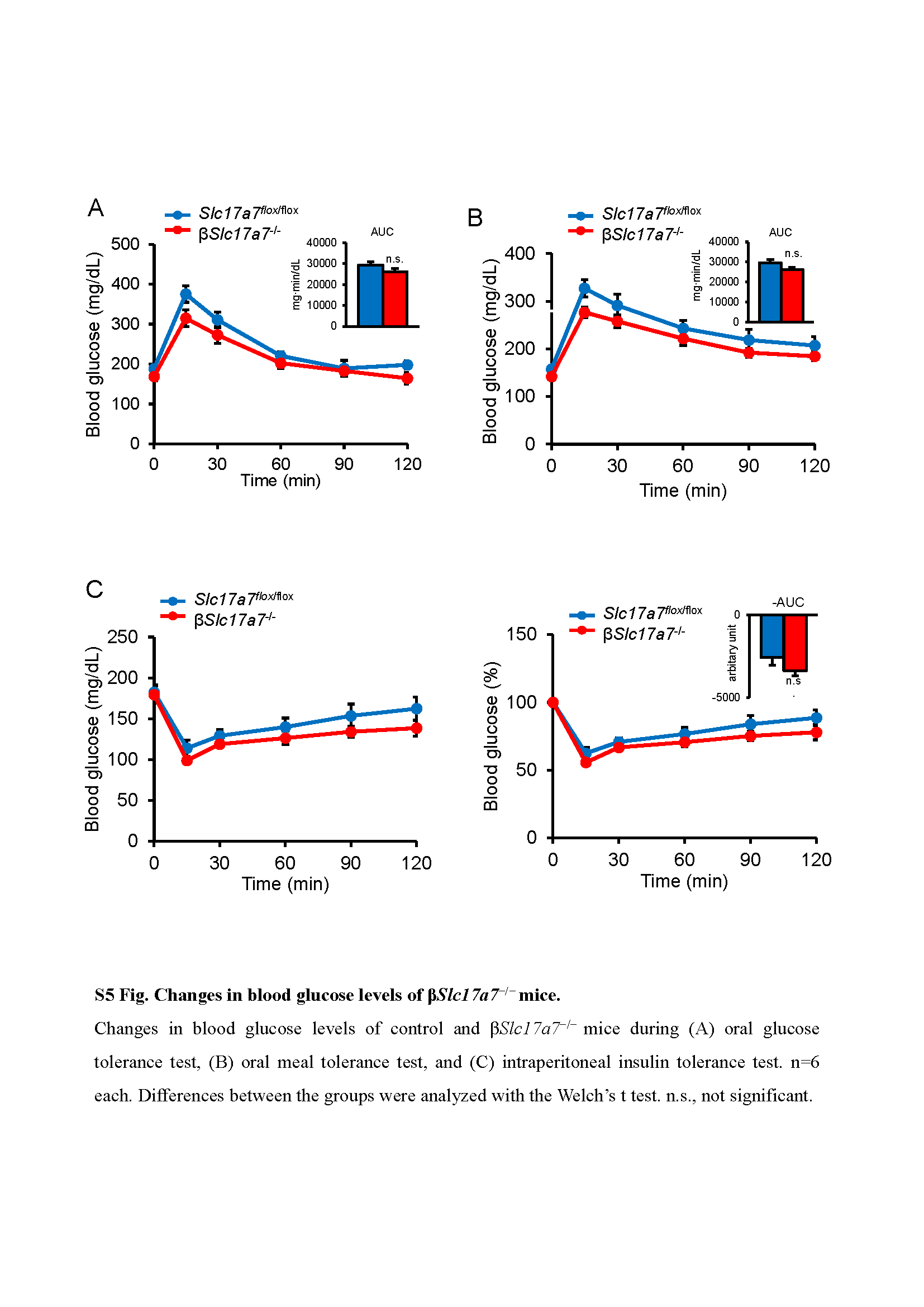

Supplement: S5 Fig — Changes in blood glucose levels of control and βSlc17a7−/− mice during (A) oral glucose tolerance test, (B) oral meal tolerance test, and (C) intraperitoneal insulin tolerance test. n = 6 each. Differences between the groups were analyzed with the Welch’s t-test. n.s., not significant. (TIFF) [file pone.0187213.s006.tiff]

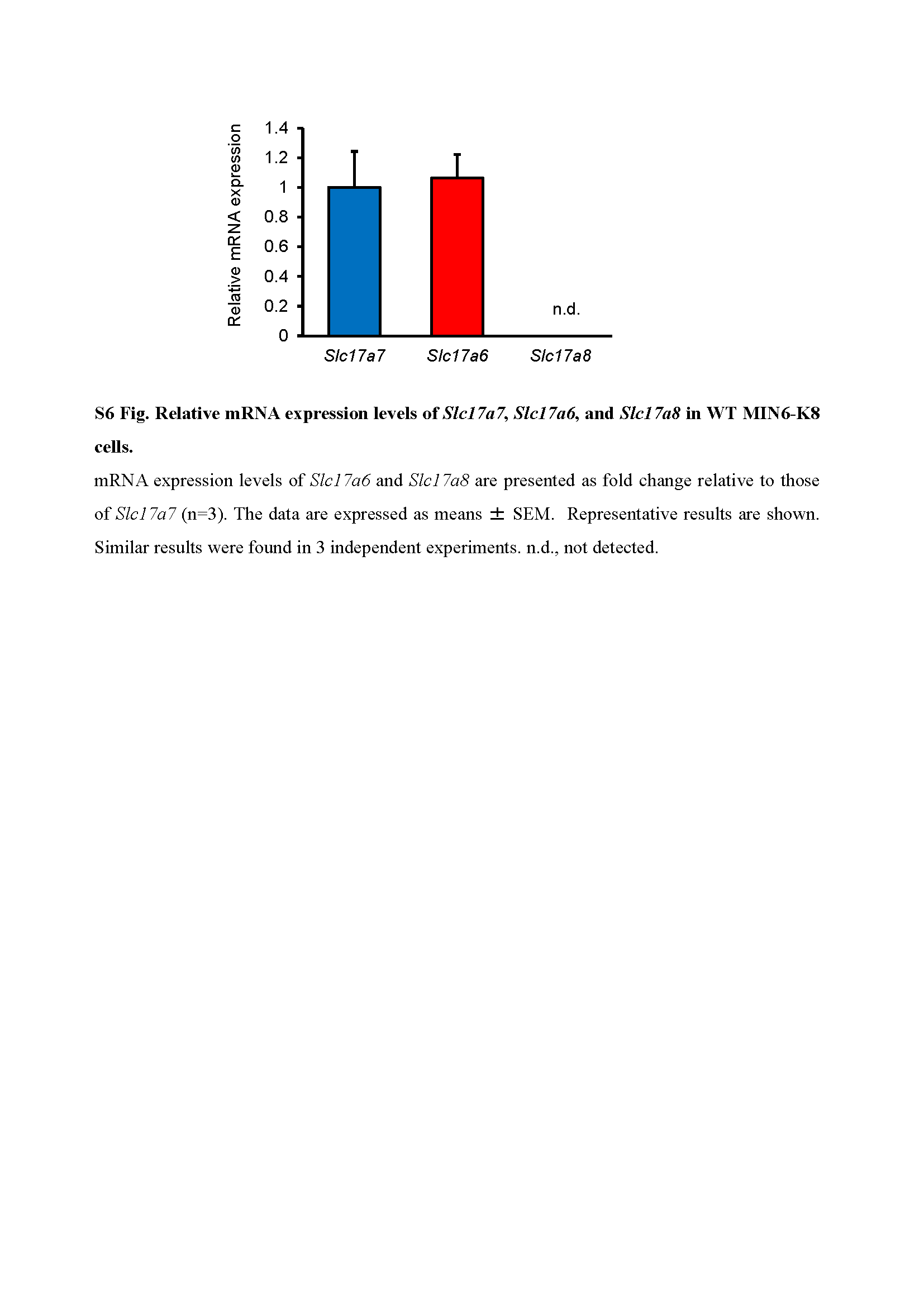

Supplement: S6 Fig — mRNA expression levels of Slc17a6 and Slc17a8 are presented as fold-change relative to those of Slc17a7 (n = 3). The data are expressed as means ± SEM. Representative results are shown. Similar results were found in 3 independent experiments. n.d., not detected. (TIFF) [file pone.0187213.s007.tiff]

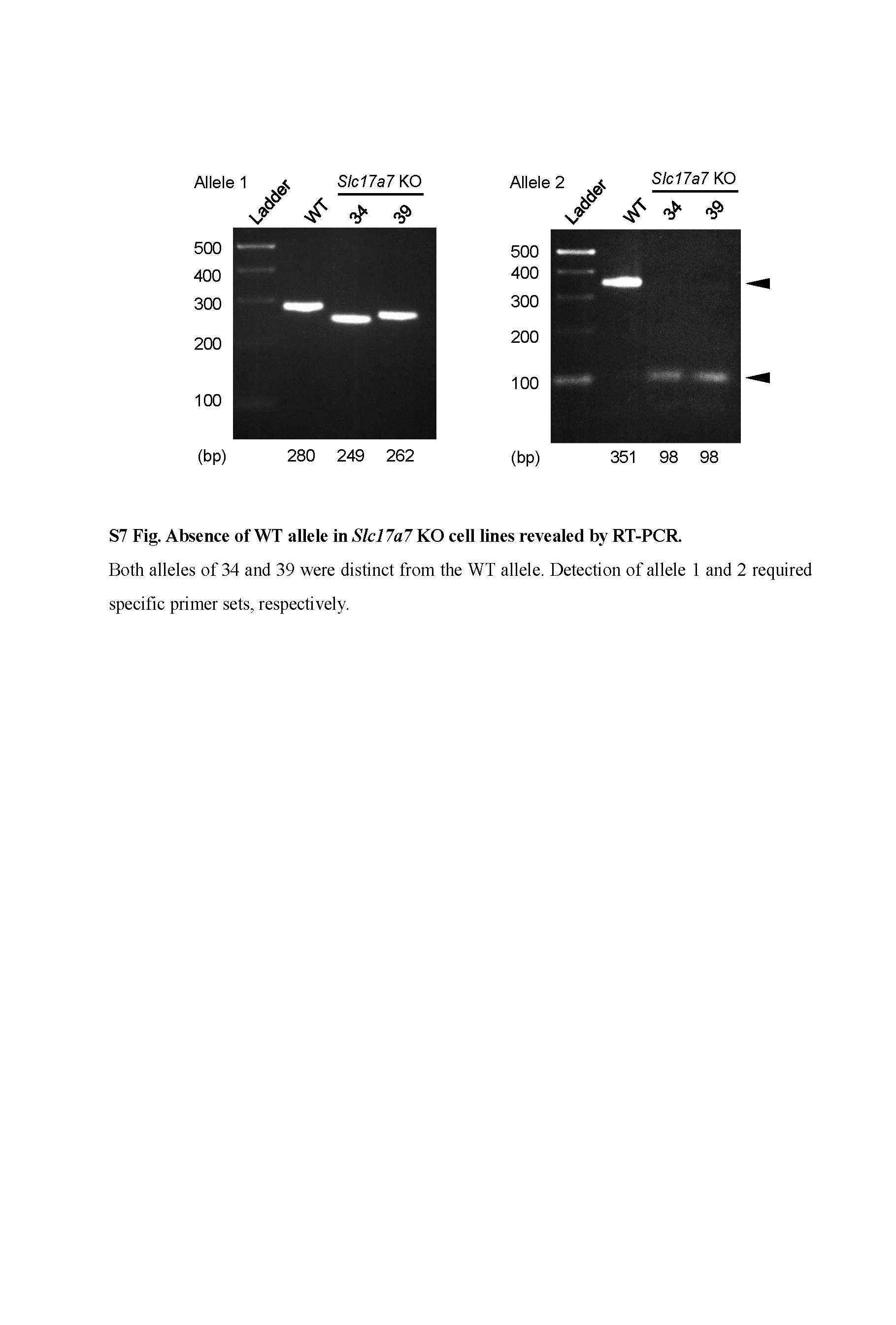

Supplement: S7 Fig — Both alleles of Slc17a7 KO cell lines 34 and 39 were distinct from the WT allele. Detection of allele 1 and 2 required specific primer sets, respectively. (TIFF) [file pone.0187213.s008.tiff]

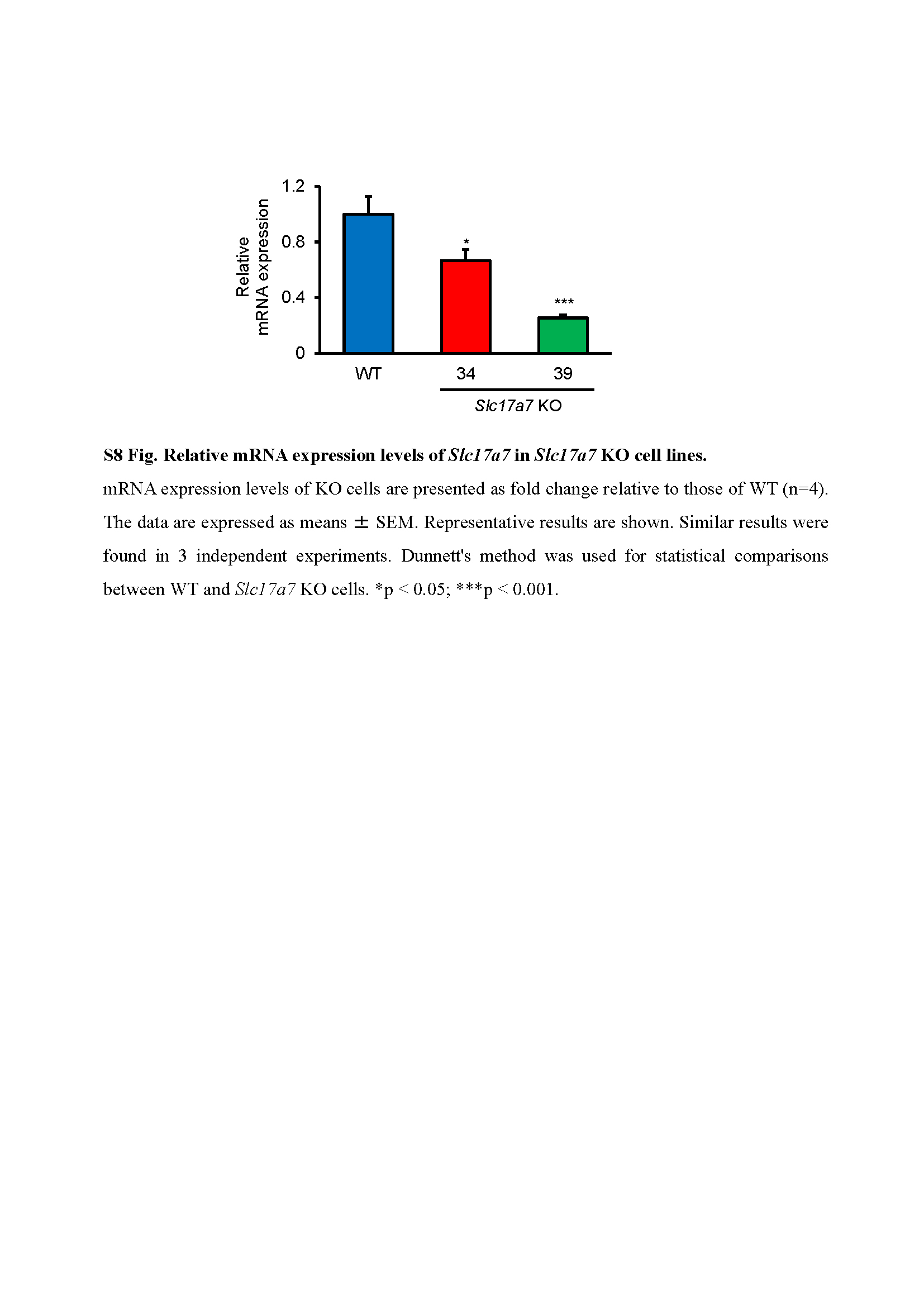

Supplement: S8 Fig — mRNA expression levels of KO cell lines are presented as fold-change relative to those of WT (n = 4). The data are expressed as means ± SEM. Representative results are shown. Similar results were found in 3 independent experiments. Dunnett's method was used for statistical comparisons between WT and Slc17a7 KO cell lines. *p < 0.05; ***p < 0.001. (TIFF) [file pone.0187213.s009.tiff]

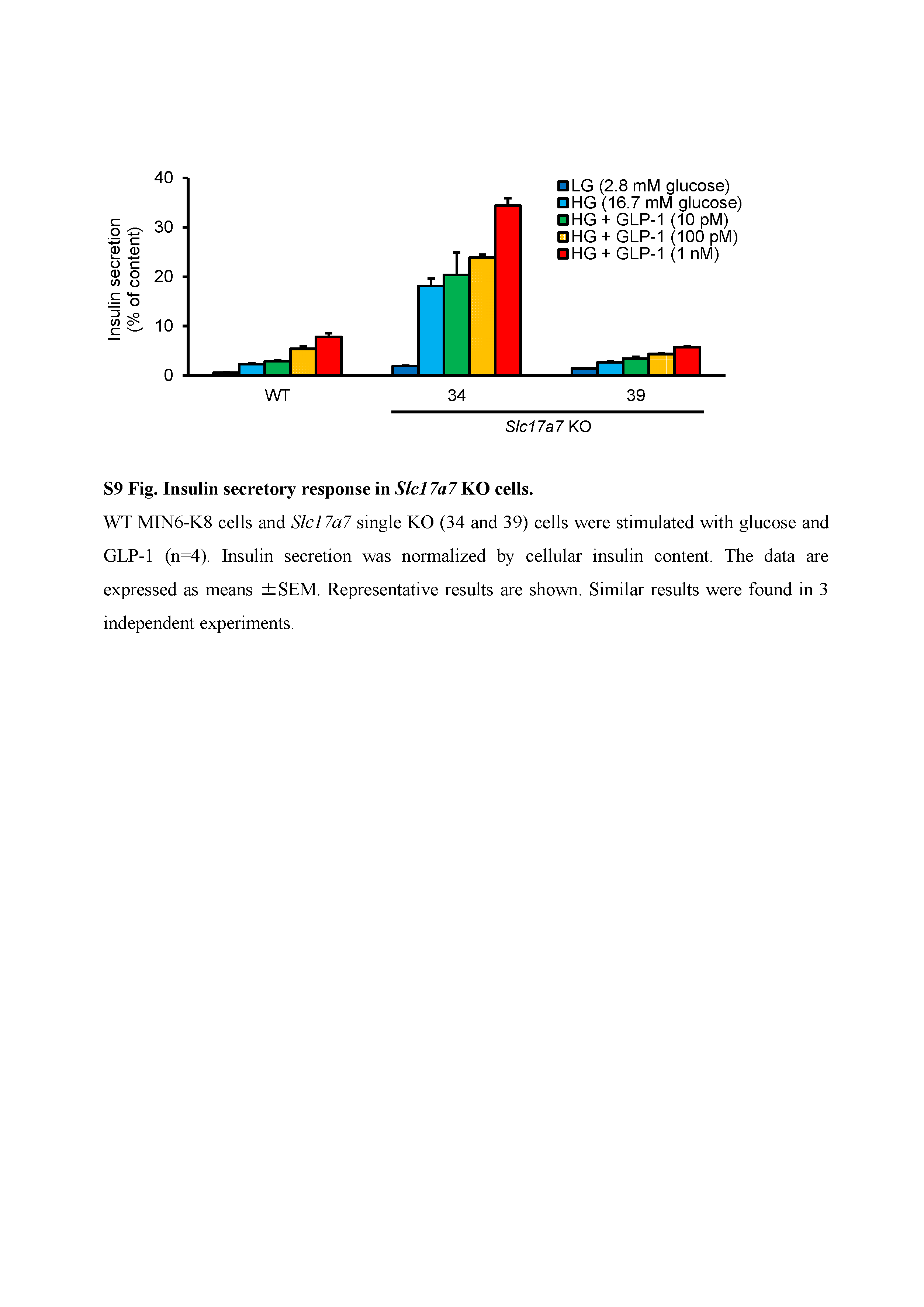

Supplement: S9 Fig — WT MIN6-K8 and Slc17a7 single KO (34 and 39) cell lines were stimulated with glucose and GLP-1 (n = 4). Insulin secretion was normalized by cellular insulin content. The data are expressed as means ±SEM. Representative results are shown. Similar results were found in 3 independent experiments. (TIFF) [file pone.0187213.s010.tiff]

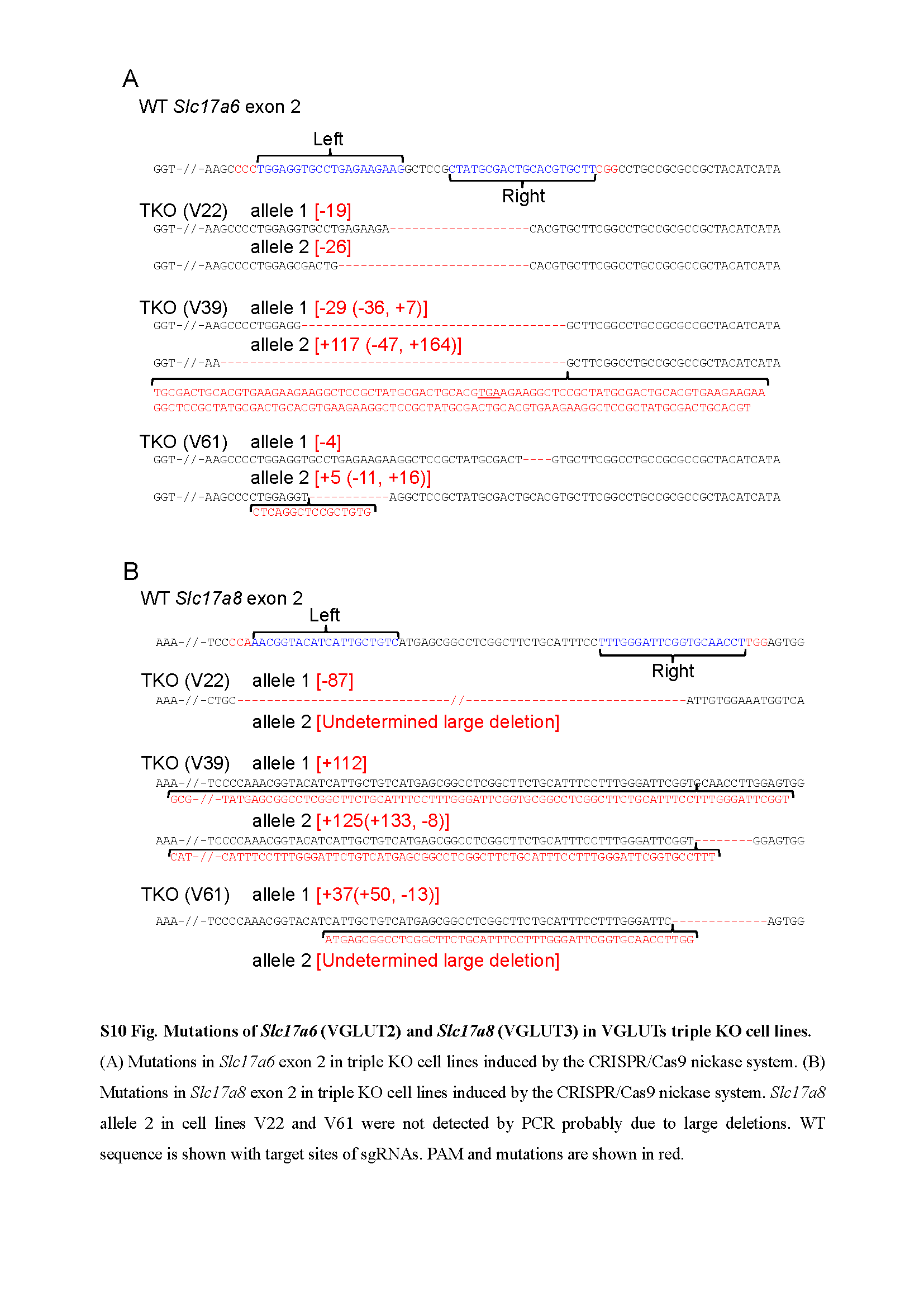

Supplement: S10 Fig — (A) Mutations in Slc17a6 exon 2 in triple KO cell lines induced by the CRISPR/Cas9 nickase system. (B) Mutations in Slc17a8 exon 2 in triple KO cell lines induced by the CRISPR/Cas9 nickase system. Slc17a8 allele 2 in cell lines V22 and V61 were not detected by PCR probably due to large deletions. WT sequence is shown with target sites of sgRNAs. PAM and mutations are shown in red. (TIFF) [file pone.0187213.s011.tiff]

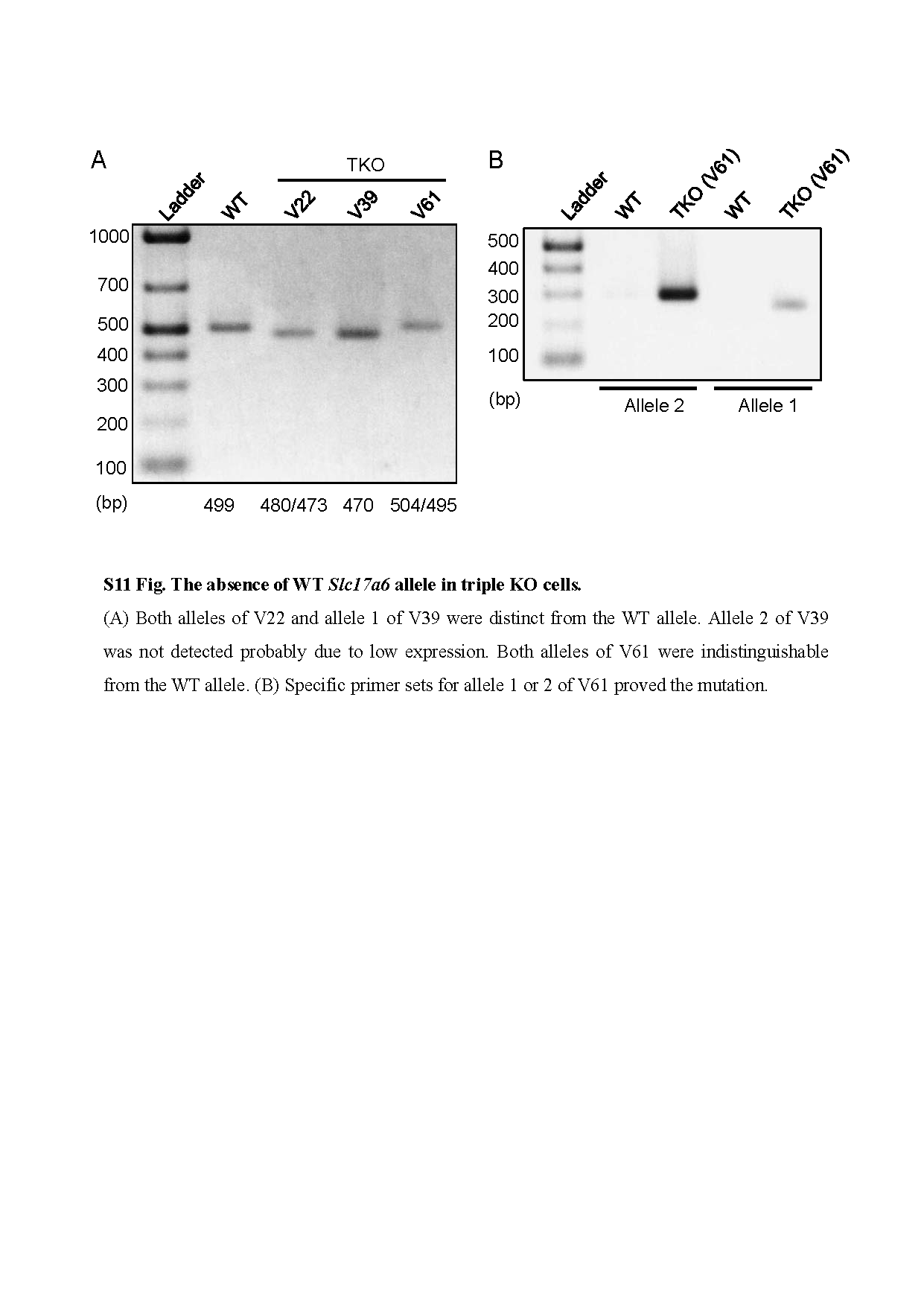

Supplement: S11 Fig — (A) Both alleles of TKO cell line V22 and allele 1 of TKO cell line V39 were distinct from the WT allele. Allele 2 of TKO cell line V39 was not detected probably due to low expression. Both alleles of TKO cell line V61 were indistinguishable from the WT allele. (B) Specific primer sets for allele 1 or 2 of TKO cell line V61 proved the mutation. (TIFF) [file pone.0187213.s012.tiff]

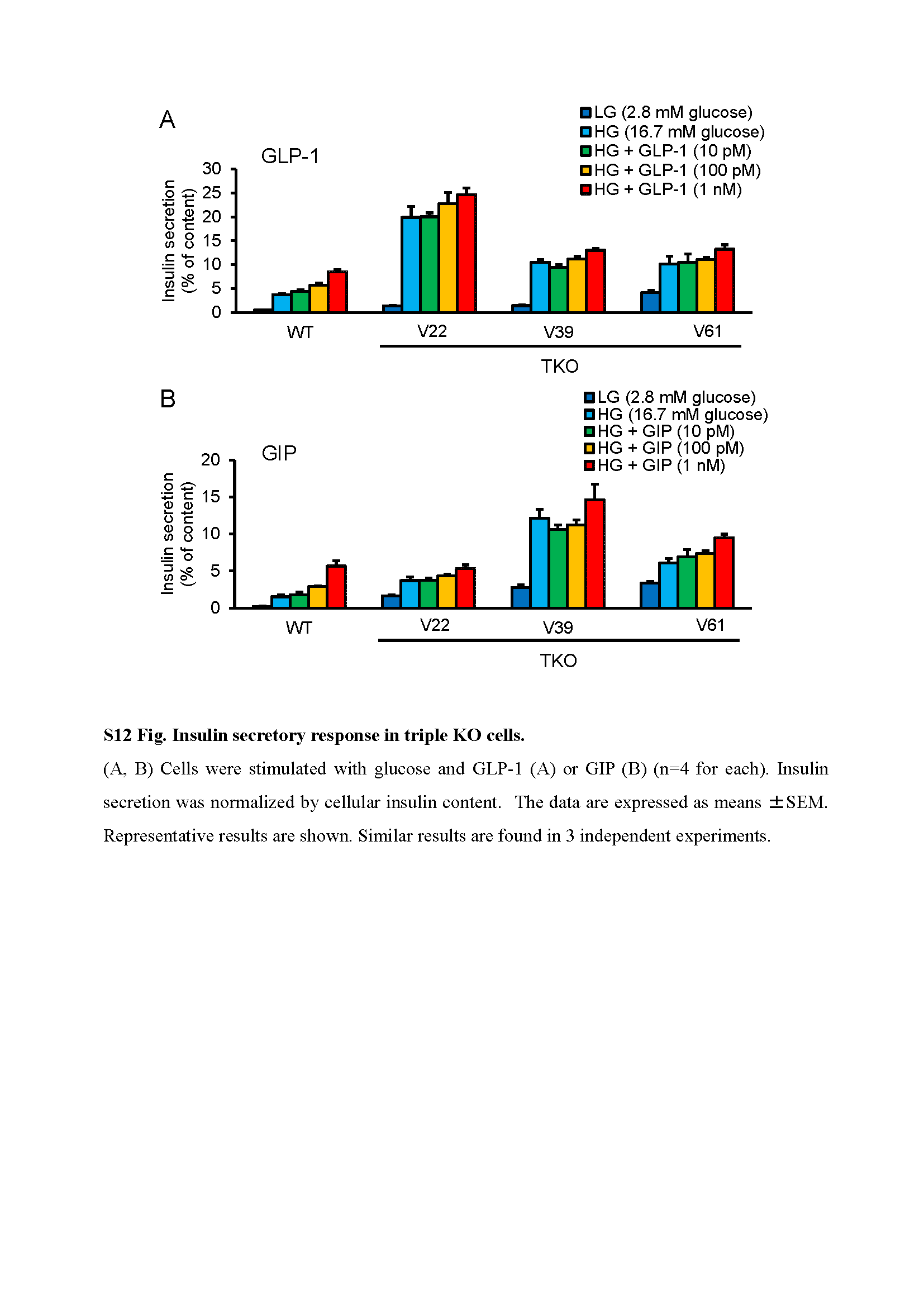

Supplement: S12 Fig — (A, B) Cells were stimulated with glucose and GLP-1 (A) or GIP (B) (n = 4 for each). Insulin secretion was normalized by cellular insulin content. The data are expressed as means ±SEM. Representative results are shown. Similar results are found in 3 independent experiments. (TIFF) [file pone.0187213.s013.tiff]
